# Editorial: Building health through physical activity in schools, volume II

**DOI:** 10.3389/fspor.2026.1770693

**Published:** 2026-01-20

**Authors:** Luís Branquinho, Pedro Forte, Ricardo Ferraz, José E. Teixeira, Andrew Sortwell

**Affiliations:** 1 Biosciences School of Elvas, Polytechnic Institute of Portalegre, Elvas, Portugal; 2 Life Quality Research Center (LQRC-CIEQV), Santarem, Portugal; 3 Research Center in Sports Sciences, Health Sciences and Human Development, Covilhã, Portugal; 4 CI-ISCE, Higher Institute of Educational Sciences of the Douro, Penafiel, Portugal; 5 Department of Sports, Higher Institute of Educational Sciences of the Douro, Penafiel, Portugal; 6 Department of Sports Sciences, Instituto Politécnico de Bragança, Bragança, Portugal; 7 Research Center for Active Living and Well-Being (Livewell), Polytechnic Institute of Bragança, Bragança, Portugal; 8 Sport Sciences Department, University of Beira Interior, Covilhã, Portugal; 9 Sport Department, Polytechnic Institute of Guarda, Guarda, Portugal; 10 Department of Sports Sciences, Polytechnic of Cávado and Ave, Guimarães, Portugal; 11 SPRINT—Sport Physical Activity and Health Research & Inovation Center, Rio Maior, Portugal; 12 School of Education, The University of Notre Dame Australia, Sydney, NSW, Australia; 13 School of Health Sciences, The University of Notre Dame Australia, Sydney, NSW, Australia

**Keywords:** active methodologies, healthy behaviors, motivation, pedagogy, physical education

Building health through physical activity in schools remains a global priority, as educational systems face persistent challenges related to declining physical activity levels, increasing sedentary behavior, and growing concerns about physical, psychological, and social health among children and adolescents. Schools represent a uniquely powerful setting to influence lifelong health behaviors, yet effective promotion of physical activity requires evidence that integrates individual, pedagogical, environmental, and structural dimensions. The 19 articles included in “*Building Health Through Physical Activity in Schools – Volume II*” collectively provide a comprehensive and multidimensional perspective on how physical activity, health, and well-being can be fostered through educational contexts across diverse populations and sociocultural settings.

## 1 Physical activity, health, and psychosocial outcomes in school contexts

At a global level, Encarnação et al. examined associations between physical activity and test anxiety among adolescents from 56 countries using PISA data, showing that higher physical activity levels are generally linked to lower anxiety, although this relationship varies substantially across cultural and educational contexts. These findings underscore the importance of context-sensitive approaches when interpreting and implementing school-based physical activity initiatives.

The psychosocial impact of school-based physical activity is further illustrated by Murillo et al., who evaluated a cycling intervention implemented in U.S. middle schools following COVID-19 lockdowns. Their results demonstrated modest yet meaningful improvements in well-being, alongside clear dose–response relationships between physical activity, sleep, screen time, and mental health outcomes, underscoring the relevance of integrated lifestyle approaches in post-pandemic school settings.

Complementing this evidence, Grasaas et al. showed that sport participation among Norwegian adolescents is associated with more favorable school-related outcomes, including lower perceived stress, reduced tiredness during school hours, and a stronger sense of belonging. Importantly, their findings also revealed pronounced socioeconomic disparities in sport participation and dropout, highlighting equity as a central consideration in school-based physical activity promotion.

## 2 Family, school environment, and structural determinants

Several contributions highlight the importance of environmental and structural influences on physical activity behavior. Dai et al. developed and validated a scale to assess school environmental factors affecting adolescent physical activity, providing a robust methodological tool to support research and policy development in educational contexts.

Using longitudinal data, Ziegeldorf et al. demonstrated that parents' educational level and Family Health Climate are strongly associated with physical activity trajectories and organized sport participation from early primary school, reinforcing the need for early, family-sensitive strategies that complement school-based initiatives.

At a broader policy level, Olivares-Arancibia et al. showed through a large-scale systematic review that sports-oriented school uniforms are associated with higher physical activity levels and improved physical fitness, particularly among girls. These findings illustrate how institutional policies, often considered peripheral, may exert meaningful effects on students' health behaviors.

## 3 Physical fitness, motor development, and movement opportunities

The role of physical activity in supporting physical fitness and motor development during childhood is addressed in several studies. Aniśko et al. identified extracurricular sports participation and body mass index as key determinants of physical fitness in school-aged children, with handgrip strength emerging as a central indicator of overall physical performance.

Webb et al. demonstrated that increased daily recess time is associated with improvements in muscular strength and neuromuscular control among young children, highlighting the value of unstructured movement opportunities and the role of physical education teachers in monitoring physical development.

At a population level, Wang et al. synthesized evidence from randomized controlled trials and confirmed that physical activity interventions produce favorable effects on anthropometric and physiological outcomes in preschool and school-aged children, reinforcing physical activity as a cornerstone of early obesity prevention.

## 4 Motor competence and after-school interventions

Extending beyond curricular physical education, Albaloul et al. examined the effects of an after-school Comprehensive School Physical Activity Program implemented among children from low socioeconomic backgrounds in the United States. While significant improvements in overhand throwing performance were observed, perceived motor competence remained unchanged, highlighting the importance of addressing both skill development and self-perception to support sustained engagement.

## 5 Pedagogy, learning processes, and engagement in physical education

Pedagogical quality and learning design emerged as key themes across several contributions. Johansen et al. provided real-world observational evidence of physically active learning, demonstrating substantial variability in physical activity intensity depending on pedagogical organization, space, and learning objectives.

Hanna et al. compared the traditional Daily Mile™ with a modified version and showed that increased variety and pedagogical flexibility led to broader improvements in motor competence, health-related quality of life, and enjoyment, supporting adaptive and student-centered program design.

Focusing on instructional sequencing, Matsuura et al. demonstrated that implementing implicit learning before explicit instruction enhances enjoyment while supporting motor skill acquisition, offering practical insights for physical education and teacher education contexts.

## 6 Motivation, physical literacy, and behavioral change

Understanding motivation and meaning is central to sustaining physical activity. Bingham et al. explored children's perceptions of physical literacy, revealing that enjoyment, social relationships, emotional well-being, and perceived competence are fundamental to meaningful movement experiences and lifelong engagement.

Ranaei et al. demonstrated that a peer education intervention grounded in the Theory of Planned Behavior effectively promoted physical activity among Iranian adolescent girls, reinforcing the value of theory-driven and gender-sensitive school-based interventions.

At the classroom level, Meinokat et al. examined physical education refusal and teachers' strategies, including the use of digital media, proposing a multidimensional framework that integrates institutional, relational, and classroom factors within increasingly digitalized learning environments.

## 7 Physical activity, health, and educational or cognitive outcomes

The complex relationship between physical activity, health, and learning outcomes is further explored in diverse contexts. Mwakalebela et al. reported high levels of physical activity among Tanzanian schoolchildren but found no direct association with cognitive performance, highlighting the multifactorial nature of cognition and the influence of contextual variables such as nutrition and socioeconomic conditions.

Chu et al. emphasized the role of sedentary behavior, rather than physical activity alone, in relation to scoliosis screening outcomes among Chinese students, underscoring the importance of addressing prolonged sitting alongside physical activity promotion.

Finally, Moravecz et al. examined health behavior socialization among teacher education students, revealing the enduring influence of family background and raising important questions about the extent to which teacher training programs can act as catalysts for long-term health behavior change.

## 8 Conclusion and future directions

Together, the 19 studies included in “Building Health Through Physical Activity in Schools – Volume II” demonstrate that promoting physical activity in educational settings requires a multilevel, interdisciplinary, and context-sensitive approach. Individual behaviors, pedagogical practices, family environments, school policies, and broader socioeconomic structures interact to shape physical activity, health, and well-being across the lifespan. Future research and practice should prioritize integrated and equitable interventions, pedagogical innovation, and policies that reduce structural barriers, while amplifying the perspectives of children, adolescents, teachers, and school communities. Educational systems thus remain central to building health through physical activity, both within and beyond the school years.

