# Correction: Editorial: Building health through physical activity in schools, volume II

**DOI:** 10.3389/fspor.2026.1792948

**Published:** 2026-02-05

**Authors:** Luís Branquinho, Pedro Forte, Ricardo Ferraz, José E. Teixeira, Andrew Sortwell

**Affiliations:** 1 Biosciences School of Elvas, Polytechnic Institute of Portalegre, Elvas, Portugal; 2 Life Quality Research Center (LQRC-CIEQV), Santarem, Portugal; 3 Research Center in Sports Sciences, Health Sciences and Human Development, Covilhã, Portugal; 4 CI-ISCE, Higher Institute of Educational Sciences of the Douro, Penafiel, Portugal; 5 Department of Sports, Higher Institute of Educational Sciences of the Douro, Penafiel, Portugal; 6 Department of Sports Sciences, Instituto Politécnico de Bragança, Bragança, Portugal; 7 Research Center for Active Living and Well-Being (Livewell), Polytechnic Institute of Bragança, Bragança, Portugal; 8 Sport Sciences Department, University of Beira Interior, Covilhã, Portugal; 9 Sport Department, Polytechnic Institute of Guarda, Guarda, Portugal; 10 Department of Sports Sciences, Polytechnic of Cávado and Ave, Guimarães, Portugal; 11 SPRINT—Sport Physical Activity and Health Research & Inovation Center, Rio Maior, Portugal; 12 School of Education, The University of Notre Dame Australia, Sydney, NSW, Australia; 13 School of Health Sciences, The University of Notre Dame Australia, Sydney, NSW, Australia

**Keywords:** active methodologies, healthy behaviors, motivation, pedagogy, physical education

Affiliations 12 and 13 were omitted for author Andrew Sortwell. These affiliation have now been added.

An incorrect Loop profile was linked to the author Andrew Sortwell. The correct link is: https://loop.frontiersin.org/people/2191389.

The original version of this article has been updated.

